# The Laboratory Diagnosis of Syphilis

**Published:** 1910-02-26

**Authors:** Hugh Wansey Bayly

**Affiliations:** Pathologist to the London Lock Hospital, Assistant in the Bacteriological Department of St. George's Hospital


### Hospital Clinics.

### THE LABORATORY DIAGNOSIS OF SYPHILIS.

By HUGH WANSEY BAYLY, M.A.Camb., M.R.C.S., L.R.C.P.; Pathologist to the London Lock Hospital, Assistant in the Bacteriological Department of St. George's Hospital.

(Read before the West London Medico-Chirurgical Society, December 3, 1909.)

It is difficult to believe that only five years ago the specific organism of syphilis was unknown, and that the morbid histologist was the only contributor to the laboratory diagnosis. The pathologist was then but very seldom consulted in this disease, whereas now syphilis ranks with tubercle, typhoid, malaria, and diphtheria as a disease in which the laboratory can afford very great help to the clinician, and can make a positive diagnosis in cases where such a diagnosis could not possibly be made with certainty by purely clinical methods.

### Antigen and Antibody.

May I first recall that by antigen is meant a substance which, when it is introduced into a living body, stimulates the cells of that body to produce an antibody, which destroys or neutralises it. It is probably by this antigen action that recovery takes place in most bacterial diseases. In the case of toxin and anti-toxin this reaction can take place directly in the body or *in vitro*, and the reaction appears to be a purely chemical one. With cells, or micro-organisms, or the extract or solution of cells, or micro-organisms, however, the action is rather more complex. The antigen and anti-body do, indeed, become linked together, but the presence of a third substance is necessary to activate or stimulate this union of antigen and antibody before the neutralising or destructive process can be completed. Various names have been given to this third activating body by different workers, but I shall only refer to it by its most familiar name—complement. The complement, in activating the mixture of antigen and anti-body becomes used up, and is, therefore, not available for further use.

Antigen and antibody are generally specific, so that a diphtheria antigen produces only a diphtheria anti-body, and a hæmolytic antigen (namely, red blood cells) produces only an anti-body to the same variety of blood cells. Complement, however, which is a normal and constant constituent of all fresh blood sera, is not specific, although probably several complements exist, and can activate any antigen united with its own antibody. Antibodies are very stable, and preserve their properties for a very long time, and are not destroyed by a temperature of 60° C. Complement, on the other hand, is unstable and is destroyed in a few days at room temperature, or in half an hour at 60° C. Fisher uses the analogy of a lock and key and hand for antigen, antibody, and complement respectively. Thus, any hand can turn any key in its own lock, but the key can only turn its own lock, and the key cannot turn in the lock without the hand.*

* *Pfeiffer's Phenomenon*, discovered in 1894, which showed the bacteriolytic action of cholera immune sera, was the starting point of the serum diagnosis of syphilis.

### Wassermann's Reaction.

The Wassermann reaction, as originally described, consists of ascertaining whether the tested serum contains any syphilitic antibody, extract of liver being used as antigen. A hæmolytic system is used for the second part of the experiment, in order to test whether the complement has been used up in the first part of the experiment or not. The specific antigen and antibody, or any other antibody present in a normal serum, can each separately absorb a small quantity of complement, but a very much greater quantity of complement is used up in activating the combined antigen-antibody, so that the specific antigen destroying reaction can occur. Thus, if one volume of complement is absorbed by two volumes of specific antigen, specific antibody, or normal serum, when acting on each separately, making three volumes in all, five, ten, or more volumes of complement will be absorbed by the combined specific antigen and antibody.

When testing for the presence of a syphilitic antibody in the test serum therefore, we must add an excess of complement over the amount that could be absorbed by specific antigen or normal serum. If the patient's serum contains no antibody, the complement will remain free, and therefore will be able to sensitise the hæmolytic system, with the result that hæmolysis occurs.

### The Method.

We will take the different ingredients in order; they are five in number: two antigens, two antibodies, and one complement. To obtain the test serum from the patient various methods are employed. I myself use a fairly large-bore needle connected with a hollow metal needle holder, to the distal end of which about six inches of fine rubber tubing is attached. The whole apparatus is sterilised by boiling for three minutes, and the blood is obtained from the most prominent vein at the bend of the elbow. The site of the puncture is previously sterilised with ether and absolute alcohol. The needle will be found to slip into the vein with greater ease if it is lubricated with some sterile vaseline. If an arm bandage is used to render a vein more prominent, on no account must the needle be removed till the bandage is loosened, otherwise the blood will, owing to the high pressure in the vein, escape through the puncture made by the needle and produce a hæmatoma. The blood is collected in a sterile tube, and, after being allowed to stand for a couple of hours, is centrifuged, and the clear serum drawn off with a sterile pipette and stored and sealed in small sterile phials. The serum is then decomplementised in a water bath at 60° C.

The syphilitic antigen is prepared by cutting and pounding a washed congenital syphilitic liver, and making either a 10 per cent. alcoholic extract or 20 per cent. watery extract. The emulsions should be shaken occasionally for 24 hours, after which they can be centrifuged, and the clear, supernatant fluid pipetted off and stored in a stoppered bottle. In the case of the watery extract .5 per cent. of carbolic acid should be added.

The hæmolytic antigen is fresh defibrinated sheep's blood. Normal salt solution is added, and the corpuscles thoroughly washed by rotating and inverting the tube once or twice without any vigorous shaking. This is again centrifuged, and the supernatant fluid pipetted off. In this way washed corpuscles, free from plasma, are obtained.

### Serum Collecting.

For hæmolytic antibody a rabbit is injected either in the auricular vein or peritoneal cavity with the washed corpuscles of from 1 increasing to 5 c.c. of fresh sheep's blood. This is repeated every four days, three injections being usually sufficient to produce a strongly hæmolytic antibody in the rabbit's serum. The rabbit is now bled from a vein in the ear, 10 c.c. being easily obtained, and the serum placed in small sterile phials, which are heated for three successive days for three-quarters of an hour at 60° C., thus ensuring sterility of the serum and destruction of all complement.

A guinea-pig, having been anæsthetised with ether, the front wall of the chest is removed and the blood collected from the heart by a sterile pipette. After standing for a short time, so as to permit of clotting, this blood is centrifuged and the serum pipetted off. This contains the complement. One small test tube is required for each serum to be examined and for the three controls. Let us suppose that there are seven sera to be examined, making a total of 10 with the three controls. The quantities that I have found by experience to be best are: 2.5 c.c. normal salt solution, .2 c.c. of liver extract (antigen), and .1 c.c. complement per each tube. Considerable time is saved, and, I think, greater accuracy obtained, if these are first mixed in bulk instead of separately in each test tube, and afterwards measured into the test tube, to which .3 c.c. of the serum to be tested is added, one serum to each tube. In this way there are only two pipette measurements instead of four, which would be necessary if saline liver extracts, complement and test serum were added separately to each tube. The tubes are now gently shaken and placed in the incubator at 37° C. for one hour and a half. A dilution of normal saline of rabbit's serum (hæmolytic antibody) is then prepared of such a strength that 1 c.c. of this dilution, when added to 1 c.c. of a 7.5 suspension of washed sheep's corpuscles, will just, and only just, produce solution of the corpuscles when incubated for five minutes at 37° C.

### Testing Sera.

For each serum to be tested, and for the three controls, 1 c.c. of 7.5 per cent. suspension of sheep's corpuscles and 1 c.c. of a suitably diluted decomplementised hæmolytic antigen are taken and mixed together in bulk. This mixture of hæmolytic antigen and antibody is called the hæmolytic system. There is plenty of time to estimate the hæmolytic power of the serum and to prepare the correct dilution, to mix the diluted serum and the suspensions of corpuscles while the tubes of liver extract, test serum, and complement are undergoing their 1½ hours' incubation. After the incubation period the tubes are removed from the incubator, and the above-mentioned 2 c.c. of hæmolytic system are added to each tube. The tubes are again shaken and replaced in the incubator until such time as complete solution of the corpuscles is produced in the control tubes containing normal serum and salt solution. When complete solution occurs in these two control tubes the test tube rack can be removed from the incubator and the tubes examined. If the test serum is from a case of syphilitic infection, a syphilitic antibody will be present in that serum, and the complement will have been used up in the first incubation period by activating the syphilitic antigen (contained in the extract of liver) that has united with the syphilitic antibody in the patient's serum. There will, therefore, be no complement left over for the second incubation period, when the hæmolytic antibody and antigen are added; no hæmolysis can therefore take place in these tubes. If, on the other hand, the test serum contains no syphilitic antibody, the complement will not be used up, but will be free to activate the hæmolytic system, and solution of the corpuscles will be obtained. A positive reaction is obtained when there is no solution of corpuscles.

### The Value of the Reactions.

The Wassermann reaction, in 9,000 cases of syphilis, shows 80 per cent. of positives. The percentage differs but slightly with different observers. I myself have determined the reaction in over 300 cases and obtained a positive reaction in 85 per cent. Only a very small percentage of non-syphilitic cases give a positive reaction. Scarlet fever has been described as occasionally giving a positive reaction; but out of 20 cases that I examined only one gave a positive reaction, and here syphilis could not be absolutely excluded. Yaws, leprosy, and trypanosomiasis have been described as frequently giving a positive reaction. I obtained a positive reaction in a few cases of pneumonia after the crisis. In no case of tubercle or cancer, and in no healthy individual, has a positive reaction been obtained. The highest percentage of positive results is obtained in untreated congenital syphilitics, practically every case giving the reaction. In primary cases, in which the lesion has been present for less than a fortnight, practically all cases are negative; about 75 per cent. of positive reactions are recorded in primary syphilis in which the lesion has been present over a month. Secondary syphilis with symptoms gives a positive result in over 90 per cent. of cases, and tertiary syphilis in about 75 per cent. of cases. Of parasyphilitic conditions general paralysis gives a positive result in 90 per cent. and tabes 50 per cent.

The general opinion seems to be that a patient who gives a positive reaction cannot be considered free of the infection, and that neither can an isolated negative result be taken as conclusive proof of the extermination of the disease, as, even in untreated cases, a negative result has occasionally been observed for a short time. In infected cases, however, these negative phases are always transitory.

### Some Results.

I have lately examined 112 cases, with a view to ascertaining the effect of anti-syphilitic treatment on the reaction. One hundred cases have received mercurial treatment either in the form of pills or injection, and the remaining 12 have received a full course of treatment with arylarsonates—soamin and orsudan being the preparations used. Only one of the cases treated with the arylarsonates gave a negative reaction at the conclusion of the treatment, and, as unfortunately I was unable to obtain a specimen of serum in this one case prior to the commencement of the treatment, it is uncertain whether the negative reaction was due to the exhibition of the drug, or whether the case had from the beginning been one of the small minority of syphilitic cases which fail to give a positive reaction. As I found in my previous series that 9 per cent. of undoubted cases of secondary syphilis failed to give a positive Wassermann reaction, I have no reason to suppose that the single negative result obtained in the 12 cases treated with arylarsonates was due to such treatment. On the other hand, the effect of mercurial treatment is clearly shown. Out of 48 cases that had received less than six months' treatment 44 still gave a positive result, four only gave a negative reaction. The proportion of negatives, therefore, was not higher than would have been expected if no treatment had been given. Of 23 cases of treatment of 6 months to one year 10 were negative and 13 positive. Of 18 cases that received treatment for 1½ years, 13 were negative and 5 positive. Of the remaining 11, all of which had received two or more years' treatment, all but two were positive; but I consider this high proportion of positive reactions in these well-treated cases due to the fact that they were not amenable to treatment; and it was probably because they were continually suffering from reminders that they continued the treatment so long. The 18 cases that received 18 months' treatment were all inmates of the Rescue Home attached to the Lock Hospital, where they remain for 15 months after leaving the hospital. With the out-patients, however, if a patient attends for over 18 months, it is usually due to the fact that occasionally symptoms arise to remind him that he is not yet free, and so a series of out-patients with 18 months' attendance means a series of abnormally severe cases, whereas the series of 18 taken from the Rescue Home were quite normal, unselected cases, and can therefore be taken as typical of cases which have received thorough treatment regulated by skilled supervision. It will be seen that 43.5 per cent. of negative reactions were recorded with treatment of six months to one year, and 72 per cent. with treatment of 18 months.

### And Conclusions.

The Wassermann reaction, though of great value for the diagnosis of secondary, tertiary, congenital, or latent syphilis, or of parasyphilitic conditions, is not of much assistance in enabling us to determine whether a sore which has just appeared is syphilitic or not; it is just in this particular class of case that we can rely with practically complete confidence on a simple, rapid, and certain method for demonstrating the living treponema pallidum in scrapings from a suspected sore or papule. By means of a disc of black enamel painted on the under surface of the condenser, all rays of light from the microscope mirror, with the exception of those at the periphery of the condenser, are cut off. Those passing through the clear periphery are deflected by suitably cut lenses so that they converge obliquely on the object examined, which appears as a bright refractive body on a dark background. In this way very transparent objects, which are invisible by direct illumination, are seen and studied. Although this valuable aid to diagnosis has been up to the present neglected by the large majority of English practitioners, yet for more than a year it has been constantly employed in Paris, and few, if any, Parisian syphilologists or dermatologists would now venture to give an opinion on a doubtful primary or secondary syphilitic lesion without previously examining a scraping for the specific organism with the help of the ultra microscope. Without doubt this method is the most practical and certain now available for ascertaining whether a lesion of the skin or mucous membrane is syphilitic or not. In Paris out of 500 recent untreated chancres only two have failed to show the treponema pallidum when examined by this method.

### Direct Microscopic Examination.

The chancre, papule, or mucous plaque should be first dried and gently rubbed with some sterile gauze to remove any superficial discharge. The margin is now gently scraped till blood just begins to exude. The surface is now again dried with some sterile gauze and a little blood or serum expressed. A small drop of this is removed with a platinum needle and mixed with a drop of distilled water on a thin glass slide. If this slide is too thick, the rays of light may come to a focus below the surface, and unsatisfactory illumination be obtained. A large cover glass is now pressed down firmly, so that only a thin layer of fluid remains between the slide and the cover glass. This can be conveniently done by spreading a piece of lint over the knee and holding the slide by each end, with the cover glass downwards, and pressing it against the knee till the superfluous fluid is squeezed out and absorbed by the lint.

A drop of immersion oil is now placed below the slide and on the upper surface of the cover glass. The slide is then placed in position on the microscope stage, so that the drop of oil on the under-surface touches the upper surface of the ultra microscope. The 1-12-inch objective is lowered into the drop of oil in the usual way.

The treponema pallidum may be recognised by its size, shape, and movements. Its characteristic appearance is that of an extremely fine silvery spiral from 5 to 25 $\mu$ in length, with very regular and closely set spirals (about seven to the diameter of a red blood disc), the distance between spirals being 1 $\mu$.

The number of spirals ranges from 5 to 25, and the extremities of the organism are pointed. It may happen that the light is only reflected from the summit of the spirals, in which case the organism will have the appearance of a chain of equidistant luminous dots, not unlike a chain of streptococci. By carefully focussing, however, this chain of dots will be seen to be a spiral.

The spirochæta refringens, spirochæta buccalis, and the spirochæta balanitidis are much larger and quite unlike the treponema.

The treponema pallidum is found below the surface, and should be sought at the margin of the lesion. It cannot be detected in the centre of an ulcerated or necrosed area where the saprophytic spirochætæ may be seen in large numbers. The organism is found in the largest numbers in mucous plaques, is constantly present in varying numbers in primary untreated chancres, and is usually detected in the papular syphilide, and only occasionally in scrapings from a recently removed or punctured enlarged syphilitic lymphatic gland. It is very seldom found in the macular or roseolar rash. The treponema has not been found in the blood by this method, except in some cases of congenital syphilis, nor in tertiary lesions.

Both general and local treatment have a marked effect on the number of treponemata found, and the organisms tend to disappear after a few weeks from the site of the primary inoculation even without treatment. If a local antiseptic has been applied, the patient should be instructed to wash it away with plain warm water, and return in a few days' time for another examination. In the absence of treatment a sore, the nature of which is clinically doubtful, can in a few moments be demonstrated to be clearly syphilitic, and so much valuable time may be saved and the annoyance and discomfort of secondary manifestations prevented.

### General Conclusions.

From the evidence before you I hope you will have been able to arrive at the following conclusions:—

1. That syphilitic infection can be diagnosed with certainty on the very first appearance of a primary sore, by means of the ultra-microscope.

2. That the earlier treatment is begun the better for the patient.

3. That a positive Wassermann reaction is, in the absence of a few diseases, pathognomonic of syphilis.

4. That the Wassermann reaction is a very useful guide to treatment, and that anti-syphilitic treatment should be continued till a succession of negative results are obtained.

5. That the blood should be examined every six months for a couple of years at least after the first negative reaction was recorded.

My thanks are due to the many authors, Continental and British, whose articles I have "commandeered" for this paper. I should like to mention especially Mr. McDonagh, from whose valuable paper on the serum diagnosis of syphilis in the September *Practitioner*, I have freely drawn. Drs. Gastou and Comendon, of Paris, have been my authorities on the ultra-microscope, and I am indebted to Dr. Gastou both for the practical knowledge of this method of diagnosis that I obtained in his laboratory, and for his kind permission to use his diagrams.

